# Intergenerational concern relates to constructive coping and emotional reactions to climate change via increased legacy concerns and environmental cognitive alternatives

**DOI:** 10.1186/s40359-024-01690-0

**Published:** 2024-04-02

**Authors:** Stylianos Syropoulos, Kyle Fiore Law, Andrea Mah, Liane Young

**Affiliations:** 1 Department of Psychology and Neuroscience, Boston College, Chestnut Hill, MA USA; 2 The Schiller Institute for Integrated Science and Society, Boston College, Chestnut Hill, MA USA; 3 Department of Psychology, University at Albany, State University of New York Albany, Albany, NY USA; 4 Department of Psychological and Brain Sciences, University of Massachusetts Amherst, Amherst, MA USA

**Keywords:** Coping, Emotions, Climate change, Legacy, Environmental cognitive alternatives

## Abstract

As the threat of climate change looms large, and we experience first-hand the impacts of rapid global warming, researchers and clinicians emphasize the need to better understand the impact of these changes on our mental health. Existing research suggests that coping with and emotional reactions to climate change can promote action to adapt to and mitigate the impacts of climate change and reduce its negative impacts to one’s mental health. In this pre-registered study (*N* = 771) we examined whether people who display extreme intergenerational concern would also constructively cope with climate change. Empirically-identified individuals showing high intergenerational concern reported more problem-focused and meaning-based coping, and less avoidant coping strategies with climate change. Further, even though they felt guilty, angry, sorrowful and isolated, these individuals also felt hopeful about the future. These effects were explained by increased concerns about one’s legacy and higher access to environmental cognitive alternatives. By instilling values that highlight intergenerational concern as a key priority, we could thus not only increase pro-climate action, but also help individuals actively and constructively cope with changes produced by climate change.

**Supplementary Information:**

The online version contains supplementary material available at 10.1186/s40359-024-01690-0.

As climate threats reach unprecedented heights [1–4], a growing consensus among researchers and clinicians underscores the pressing need to expand our perspective beyond physical environmental repercussions and recognize the impact of the ongoing climate crisis on the mental health and well-being of our global community [5–11]. Emerging research in this vein underscores the impact of climate change on mental health, but also that coping strategies and emotional reactions strongly predict pro-environmental engagement [12, 13]. Yet, the effects of climate change are projected only to intensify over time, with its most substantial consequences expected to profoundly shape the distant future of humanity [14–16]. The growing social movement and ethical philosophy of longtermism explicitly advocates for protecting the welfare of future generations [17–19], and nascent psychological inquiry into longtermism beliefs highlights that individuals with a strong sense of intergenerational concern tend to exhibit heightened pro-environmental attitudes and engagement [20–24].

Drawing upon existing theory and evidence, we investigate whether individuals showing a high amount of intergenerational concern (1) cope with climate change more constructively and (2) emotionally respond with both more concern and hope relative to general population controls. We also explore whether legacy concerns [25] and the ability to generate cognitive alternatives to the current environmental status quo [26] mediate any noted effects of intergenerational concern on coping and emotional reactions to climate change.

## Measuring intergenerational concern using the longtermism beliefs scale

Longtermism refers to an ethical philosophy advocating equal concern for current and future generations [17–19, 27, 28] and a social movement with a modest but dedicated and rapidly expanding following [29–31]. Longtermist ideology is guided by three key principles: (1) expressing concern for the well-being of future generations, (2) acknowledging the vast potential of humanity if extinction risks are reduced, and (3) recognizing the current generation’s capacity to shape a positive future for humankind. Thus, longtermism is very much synonymous with showing a large degree of intergenerational concern.

Despite the longtermism *movement* being subject to numerous criticisms in the popular press [29, 32], the longtermism *philosophy* provides a structured framework for studying the psychological dynamics of intergenerational concern. Emerging research in this vein from social psychology and cognitive science has employed the Longtermism Beliefs Scale (LBS) [22], which leverages items derived directly from eminent philosophical writings concerning longtermist principles [18, 19] to better understand the psychological antecedents and consequences of endorsing them. A significant portion of this research employs a scoring approach to categorize participants into two distinct subject groups: “longtermists,” who show substantial levels of intergenerational concern for both near and distant future generations, and general population controls, who either do not show above average levels of intergenerational concern or exhibit a declining level of concern for future generations as the timeframe under consideration extends further into the future [22–24, 33, 34]. In essence, empirically classified longtermists show a marked departure from the prevailing tendency displayed by most individuals [35–39] to consistently undervalue the well-being of future generations.

## The psychological and pro-environmental profiles of intergenerational concern

Longtermists empirically identified through the LBS exhibit not only a greater likelihood of self-identifying with the longtermism philosophy, but also a robust profile characterized by intergenerational concern, prosocial tendencies, and farsighted attitudes and behaviors [22, 33]. For instance, longtermists exhibit greater moral consideration for the welfare of individuals in distant future generations and also extend their moral concern to present-day outgroups relative to controls, ascribing to them elevated human-like capacities and characteristics [20, 24]. To further corroborate the connection between longtermism beliefs and prosocial tendencies, we can turn to the literature on moral expansiveness and dehumanization. A substantial body of literature has established a relationship between possessing a broad moral circle and reduced dehumanization tendencies with prosocial inclinations that surpass the typical parochial biases often constraining prosociality in the broader population [40–46]. Relatedly, longtermists demonstrate heightened levels of expansive altruism that extend across social distance and elevated utilitarian decision-making. Longtermist personality profiles are characterized by patterns of traits on the BIG-5 and HEXACO inventories [47, 48] as well as on measures of Dark Tetrad traits [47, 49, 50] and Primal World Beliefs [51], which are strongly aligned [52, 53] with prosocial behavior [22–24, 33, 34]. In terms of behaviors, longtermists, when compared to members of the general population, tend to make larger donations to charities benefitting future generations, show more support for farsighted public policies, and are more inclined to invest cognitive effort for the betterment of future generations [22, 34].

However, beyond demonstrating consistent patterns of heightened intergenerational and present-day prosocial attitudes, beliefs, and behaviors, longtermists also exhibit consistent patterns of pro-environmental attitudes and both top-down and bottom-up pro-environmental engagement. Compared to non-longtermists in the general population, individuals identified as longtermists through high scores on the LBS display stronger support for pro-environmental public policies in general and initiatives advocating climate justice for future generations and present-day minoritized groups more specifically [21]. They additionally report heightened threat perceptions of issues related to climate change, participate in more pro-climate actions in their daily lives, and contribute larger donations to pro-environmental causes in the laboratory [22, 23]. Similar to how they extend moral regard across social and temporal boundaries, longtermists extend greater moral consideration to entities within the natural environment as well, including non-human animals, rainforests, and coral reefs [20, 24], providing further support for the connection between substantial levels of intergenerational concern and pro-environmentalism.

The pro-environmental tendencies of longtermists are, in part, explained by their increased ability to envision a more sustainable future and generate a greater variety of environmental alternatives compared to non-longtermists [21]. These factors are closely linked to pro-environmental engagement in the broader population as well [26, 54–56]. These findings may reflect a heightened imaginative capacity in longtermists to transcend the here and now and more vividly represent distal futures in the mind’s eye (see Episodic Future Thinking [57–60]). Moreover, longtermists exhibit elevated concerns about their own futures, specifically with regard to leaving behind a positive legacy [20–22, 34], another factor long recognized as a crucial motivator for environmental action [25, 35, 36, 61–63].

Longtermist beliefs (i.e., endorsing a sense of impartial intergenerational beneficence) and pro-environmental behavior may be informed by extant literature on generative concern. People who are worried about the well-being of future generations are considered to be high in generative concern [64, 65]. Researchers have linked generative concern to environmentalism, finding that emerging adults who score high on measures of generative concern at ages 23 and 26 reported stronger environmental identity, as well as greater pro-environmental attitudes and engagement in pro-environmental behavior at age 32 [64]. Further, when these emerging adults exhibited increased growth in generative concern between ages 23–32, they were more likely to share narratives about their environmental identity which were coded as being more personally meaningful, detailed, and having a stronger influence on their commitment to continued environmental involvement [66]. In other work, people who scored high in generativity were also more likely to report environmental concern and green purchasing behavior [67]. However, generativity, as measured in these studies and other work on generative concern using the Loyola Generativity Scale (LGS [65], ), can be distinguished from longtermism because it includes more than intergenerational concern. The LGS contains sixteen items, but only three of these items measure beliefs about the impacts one can have on others after their death (i.e., in the long term), along with other items measuring commitments to others, creativity, and desire to mentor children [65]. Thus, while we expect that longtermism will exhibit similar relationships to environmentalism as generativity, studying the specific influence of intense intergenerational concern is important.

However, despite extensive research examining the connection between intergenerational concern and climate-related attitudes and actions, a crucial and unanswered question remains: Do individuals with substantial levels of intergenerational concern cope with and emotionally react differently from members of the general population to escalating threats associated with climate change? The theoretical foundations underlying and emerging research into the psychology of longtermism hints at this possibility. For instance, longtermists actively take actions in their lives to (1) combat climate related threats [21, 34] and (2) safeguard the welfare of future generations [20–24, 34]. These findings allude to the possibility that longtermists may employ more proactive rather than avoidant coping strategies when dealing with climate change-related distress, taking practical steps to manage and mitigate the source of their concerns [5–7, 9].

Likewise, longtermists’ elevated pro-environmental engagement may be owed in part to their emotional reactions to climate change. For instance, it’s plausible that longtermists may encounter heightened negative emotions such as anxiety, anger, or guilt (see [8, 68]), when faced with climate-related challenges and when reflecting on the legacies they will leave behind. These negative emotions, in turn, could serve as a driving force behind their proactive engagement and action-oriented approach to addressing climate-related issues. Yet, longtermists also have the capacity for elevated optimism when looking towards the future, as evidenced by their ability to envision more sustainable environmental alternatives [69]. This observation could provide insight into why potentially negative emotional responses to climate change do not lead to hopelessness or avoidance, but instead drive action among individuals with substantial levels of intergenerational concern. Nonetheless, since a comprehensive study directly comparing the climate change-related coping mechanisms and emotional responses of longtermists with those of the general population has not yet been conducted, these questions remain unanswered and warrant investigation. The extensive body of literature on coping strategies and emotional reactions in the context of climate change serves to supplement what is known from the burgeoning literature on intergenerational concern and longtermism beliefs in guiding the present research.

## Coping with and emotional reactions to climate change

Coping refers to the different behavioral and cognitive strategies people use to deal with stress [70]. When we refer to coping with climate change, we are referring to the ways that people who feel worried about climate change respond to those feelings– whether they ignore the problem, express denial, and discount the threat (avoidant coping) or try to find ways to tackle the problem and directly engage with the issue (active coping). While we can broadly categorize coping strategies as being avoidant or active, researchers also describe coping based on its focus (e.g., meaning-focused, problem-focused, emotion-focused [70],. When we consider the stressors associated with climate change and the scale of the issue, it has been argued that successful coping will likely include a mix of strategies [71]– some strategies involving proenvironmental engagement, and some that help people to cope with their own emotional responses to the existential threat of climate change. For example, Ojala [9] found that, among youth, engaging in problem-focused active coping was associated with greater negative affect, but this relationship was attenuated when the youth also engaged in meaning-focused coping (e.g., positive reframing). The ways that people choose to cope with the threat of climate change have important implications for their own well-being and the environment. While it is important for people to find ways to manage their emotional responses to climate change, it is also necessary for people to take the actions that they can to address climate change through their own behavior [71].

While work on the link between coping strategies to proenvironmental behavior is sparse, there is some evidence that people who report coping actively with climate change also report greater engagement with proenvironmental behaviors [72]. However, past work has also found that people who report using more avoidant strategies also engage in proenvironmental behaviors. As long as people are saying they are coping in some form, greater concern translates to behavior [72]. Ojala [73] found that adolescents who report greater meaning-focused coping are more likely to report greater pro-environmental behavior, and that those who reported more de-emphasizing coping (e.g., telling themselves the problem is over-exaggerated) were significantly less likely to report proenvironmental engagement. Although more research is needed on the link between coping and proenvironmental behavior, particularly work measuring proenvironmental behavior via observation, it seems likely that people who report greater coping with climate change, especially in more approach-oriented ways, are more likely to engage in proenvironmental behavior as a means managing their stress.

For longtermists, we might expect that concern about climate change, combined with propensity for prosociality, may lead to active forms of coping. Longtermists, namely, individuals who are exceptional in their intergenerational concern, feel a sense of responsibility to future generations; thus, we would expect that avoidant forms of coping, like denial or disengagement with the issue, would be contrary to their values. In previous work, people who express greater concern for their legacy–having an impact on the future beyond their lifespan–tended to report greater engagement in active forms of coping with climate change, and more proenvironmental behavior [74]. These findings would lend support to a prediction that longtermists would be more likely to engage in active coping with climate change-related stress, because longtermists strongly endorse legacy motives [20–22, 34].

Another aspect of longtermism is the ability to generate environmental cognitive alternatives (ECAs). Having a sense of hope and optimism about the future and being able to imagine the possibility of humankind adapting to climate change is likely necessary to maintain long-term engagement with the issue of climate change and also to maintain personal well-being [54, 56]. Indeed, being able to make meaning in spite of the magnitude of climate change challenges attenuates the negative relationship between problem-focused coping and well-being [73]. People who express greater motivation to leave a positive legacy also report a greater sense of constructive hope about climate change, and less hope based in denial [74]. Thus, we would expect that, because they feel a greater sense of hope and less powerlessness, longtermists will be better able to generate ECAs than controls. It is likely also the case that people who are better able to imagine an alternative future in turn feel more power to achieve that future and thus feel greater hope.

Finally, the stress and concern that people feel about climate change may take different forms: grief, anger, guilt and worry. While some level of emotional response is likely needed to motivate engagement with climate change, there is also evidence that, if these emotions are too strong, it could be maladaptive [75]. For example, eco-anxiety is associated with more pro-environmental behavior, but poorer mental health [76]. Anger often motivates collective action generally [77], and this has also been found when measuring eco-anger [78]. There are many different kinds of guilt a person can feel associated with climate change (e.g., a sense of collective guilt due to feeling complicit in a system that causes climate change vs. criticism of one’s actions [79],. Inducing guilt about climate change can motivate action [80]. However, it has been theorized that, for certain people, feelings of guilt underlie a propensity to deny human-caused climate change [81]. Longtermists may feel both more anger about climate change–a perceived failure to safeguard the future–and more guilt from feeling complicit in the systems which cause climate change.

Moreover, sadness about climate change, sometimes termed ecological grief, takes many forms [82]. One form of this grief is associated with concerns about anticipated losses, those that have not yet occurred but that are likely to without action [82, 83]. For longtermists, it is likely that one of the feelings they have about climate change is a sense of loss and grief for the future, as long as they believe it poses tangible existential risk. While we might expect that feelings of sadness and depression around climate change could lead to withdrawal from or avoidance of the issue, some research suggests that people who express greater eco-depression also engage in collective action around climate change [78]. One of the objectives of the present investigation is to better understand to what extent longtermists express each of these kinds of emotions.

## Current studies

Building upon the aforementioned theoretical rationale and existing findings [20–24, 34], we hypothesize first and foremost, that longtermists will report significantly higher legacy concerns (H1), and a greater ability to generate environmental cognitive alternatives (H2), compared to non-longtermists in the general population. In addition, we hypothesize that, because climate change is itself an extinction risk, longtermists will feel more angry about inaction for climate change (H3a), less climate contempt–feelings of disregard for the issue of climate change (H3b), more hope/enthusiasm that climate change can be addressed (H3c), less powerlessness about the scope of the threat (H3d), more guilt about not doing enough to address climate change (H3e), as well as more anxiety resulting from (H3f), and sorrow regarding (H3g) climate change.

Importantly, we have varied predictions regarding how longtermists versus general population controls will score on how isolated they feel from others regarding their opinion about climate change. On the one hand, it’s possible that longtermists may, on account of climate change representing an extinction threat, perceive their care regarding climate change as being higher than average, and consequently feel greater isolation. On the other hand, longtermists may overestimate normative alignment with their own climate change attitudes and feel less isolation compared to general population controls as a result.

Finally, because longtermists are more concerned about and more likely to take active steps to address climate change [21, 34], and because they seek to protect future generations [20–24, 34], we hypothesize that they will score higher on problem-focused (H3h) and meaning-focused (H3i) coping and lower on avoidant coping with climate change (H3j).

Extending this hypothesis further, we expect that higher scores in legacy concerns and ability to generate environmental cognitive alternatives will relate to more climate change-related anger (H4a-H4b), less contempt (H5a-H5b), more climate hope/enthusiasm (H6a-H6b), less climate powerlessness (H7a-H7b), more climate guilt (H8a-H8b), more climate anxiety (H9a-H9b), more problem-focused coping (H10a-H10b), more meaning-focused coping (H11a-H11b), and less avoidant-coping (H12a-H12b). Finally, because of H1-H12, we also expect significant indirect effects of longtermism matching the aforementioned directional associations on climate emotions via higher legacy concerns and environmental cognitive alternatives as simultaneous mediators (H13). These hypotheses, together with all analytical decisions, were pre-registered on AsPredicted, https://aspredicted.org/JPK_Q37. All survey materials, data, and code for all analyses can be found on the Open Science Framework, https://osf.io/ndqz2/?view_only=adef6f1b8aa44aeeac90645feb2309f4.

## Method

All experimental protocols were approved obtained IRB approval by Boston College Institutional Review Board, Protocol #12.064.01. All participants were informed about the study and then consented to participating in it. This was completed at the beginning of the survey.

### Participants

A total of 800 participants were recruited via Prolific, an online platform that allows participants to complete surveys in exchange for financial remuneration. Per our pre-registration protocol we removed participants who had duplicate IP addresses (*N* = 12) and participants who missed our attention check (a multiple choice question asking them to select a particular response; *N* = 17). A total of 771 participants remained (*M*_age_ = 40.51, *SD*_age_ = 13.45), of whom 385 (49.9%) were male, 369 (47.9%) female, 17 (2.2%) neither male nor female, 565 (73.2%) white, 116 (15.0%) Black or African American, and 38 (4.9%) Asian. The average perceived socioeconomic status (SES) was 5.02 (SD = 1.76) on a scale of 1–10, and the average political ideology was 3.29 (SD = 1.78) on a scale of 1–7, with higher scores reflecting stronger conservatism.

### Materials and procedure

Measures were grouped based on their role (predictor, mediators, outcomes) and shown to participants in a randomized order.

#### Predictor: longtermism beliefs

Longtermism beliefs were captured with the 28-item (*a* = 0.97) Longtermism Beliefs Scale [22]. The scale consists of seven statements, which are repeated with reference to four different timeframes/timepoints (1,000, 10,000, 100,000, and 1,000,000 years in the future). Scores are captured on slider scales ranging from 0 = strongly disagree– 100 = strongly agree. Per the proposed methodology, participants are classified as longtermists if they score above 75 for the closest temporal timeframe (1,000 years), and they have the same score, or higher for future timeframes (i.e., 10,000, 100,000, and 1,000,000 years in the future).

#### Mediator: legacy concerns

Legacy concerns were captured using three items from Zaval and colleagues [25], measured on 7-point Likert scale (*a* = 0.89).

#### Mediator: environmental cognitive alternatives

Environmental cognitive alternatives (ECAS) were captured with 10 items (*a* = 0.93) from Wright and colleagues [26] measured on a 7-point Likert scale.

#### Outcome: climate change coping

Three different methods of coping with climate change were measured. These were problem-focused coping (*a* = 0.90), meaning-focused coping (*a* = 0.79), and avoidant coping (*a* = 0.87). These measures were taken directly or adapted from Ojala [9]. Each method of coping was measured with 5 items, each measured on a 7-point Likert scale. The full set of items can be found in the SOM.

#### Outcome: climate change emotions

Our goal was to capture a broadest possible array of emotional reactions to climate change, including both constructive emotional reactions with positive valence (e.g., hope) as well as reactions with a more negative valence (e.g., contempt). To that end, we utilized a tool that was recently developed by researchers to comprehensively assess the full breadth of possible emotional reactions to climate change (see [8]). Specifically, we measured climate change-related anger (*a* = 0.96), contempt (*a* = 0.94), hope/enthusiasm (*a* = 0.93), powerlessness (*a* = 0.84), guilt (*a* = 0.95), anxiety (*a* = 0.92), sorrow (*a* = 0.95), and isolation (*a* = 0.91), which span the spectrum of affective valence. Participants responded to four items per emotion on a 7-point Likert scale.

## Results

All analyses were conducted in SAS. Correlations were estimated with the *proc corr* command, t-tests with the *proc ttest* command, regression with the *proc reg* command, and indirect effect tests using the PROCESS Macro, with 10,000 bootstrapped samples.

### Correlations

As hypothesized (see Table 1) higher longtermism beliefs related to increased legacy concerns and ECAs. Each of these three measures related to decreased avoidant coping, increased problem-focused and meaning-based coping with climate change. Coping styles that were actively acknowledging climate change and focused on ways to address the issue rather than avoid it, Longtermism beliefs, legacy concerns and ECAs related to increased hope, anger, guilt, anxiety, sorrow, isolation and decreased contempt. No significant association was found with powerlessness except for ECAs, which negatively related to powerlessness.

**Table 1:** Pearson’s correlations between all measures

	1	2	3	4	5	6	7	8	9	10	11	12	13
1. Longtermism Beliefs	--												
2. Legacy concerns	0.39^**^	--											
3. ECAS	0.35^**^	0.34^**^	--										
4. Problem Focused Coping	0.48^**^	0.48^**^	0.53^**^	--									
5. Avoidant Coping	-0.33^**^	-0.11^*^	-0.16^**^	-0.38^**^	--								
6. Meaning-Based Coping	0.32^**^	0.35^**^	0.47^**^	0.55^**^	-0.05	--							
7. Anger	0.39^**^	0.22^**^	0.30^**^	0.57^**^	-0.61^**^	0.21^**^	--						
8. Hope	0.42^**^	0.38^**^	0.51^**^	0.60^**^	-0.33^**^	0.72^**^	0.45^**^	--					
9. Powerlessness	0.09	0.04	-0.12^**^	0.09	-0.04	0.00	0.33^**^	0.07	--				
10. Guilt	0.34^**^	0.28^**^	0.24^**^	0.51^**^	-0.22^**^	0.26^**^	0.50^**^	0.38^**^	0.41^**^	--			
11. Isolation	0.21^**^	0.23^**^	0.26^**^	0.38^**^	0.00	0.17^**^	0.33^**^	0.20^**^	0.30^**^	0.47^**^	--		
12. Anxiety	0.42^**^	0.29^**^	0.31^**^	0.62^**^	-0.52^**^	0.30^**^	0.74^**^	0.47^**^	0.43^**^	0.61^**^	0.45^**^	--	
13. Sorrow	0.41^**^	0.26^**^	0.29^**^	0.59^**^	-0.63^**^	0.25^**^	0.78^**^	0.46^**^	0.36^**^	0.51^**^	0.34^**^	0.78^**^	--
14. Contempt	-0.34^**^	-0.17^**^	-0.26^**^	-0.46^**^	0.81^**^	-0.17^**^	-0.63	-0.44^**^	-0.11^*^	-0.32^**^	-0.12^*^	-0.59^**^	-0.65^**^

Note.^*^p <.01, ^**^p <.001

### Differences based on longtermist identification

Of the 771 participants, a total of 185 (24%) scored in the longtermist pattern, showing high intergenerational concern. Specifically, longtermists (*M* = 92.78, *SD* = 6.34) scored significantly higher (*t*(761.73) = 41.89, *p* <.001, *d* = 2.63) than the rest of the sample (*M* = 48.87, *SD* = 22.73) in longtermism beliefs, suggesting that they were considerably higher in their perception that future people deserve moral rights and that we can influence their lives. All results were robust to the inclusion of age, gender, socioeconomic status and political ideology as covariates (i.e., results remained significant and in the same direction). For these analyses, see the Supplementary Online Materials (SOM).

#### Mediators

**Fig. 1:**
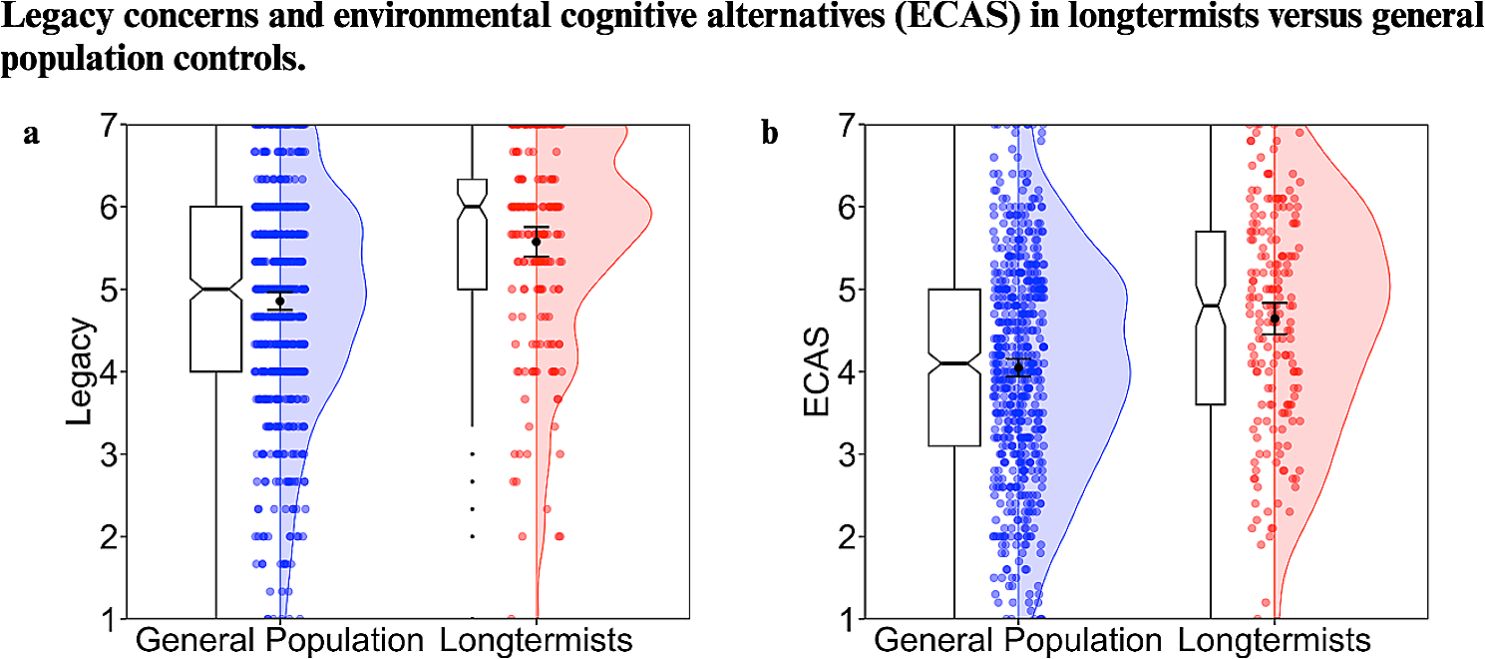
Plots depicting legacy concerns (**a**), and environmental cognitive alternatives (**b**) for longtermists and general population controls. Ratings were made on a scale from 1 to 7 and averaged to form composite measures. Colored dots correspond to individual data points and are jittered for readability, with split violin plots overlaid to show the relative distribution of scores across populations. Error bars depict ± 1.96*SEM. Notched boxplots are included, with notches depicting a confidence interval around the median with a value of +/- 1.58*IQR/sqrt(n)

Supporting our hypothesis, longtermists scored significantly higher in legacy concerns and ECAS compared to non-longtermists (see Table 2; Fig. 1).

#### Climate change emotions

**Fig. 2:**
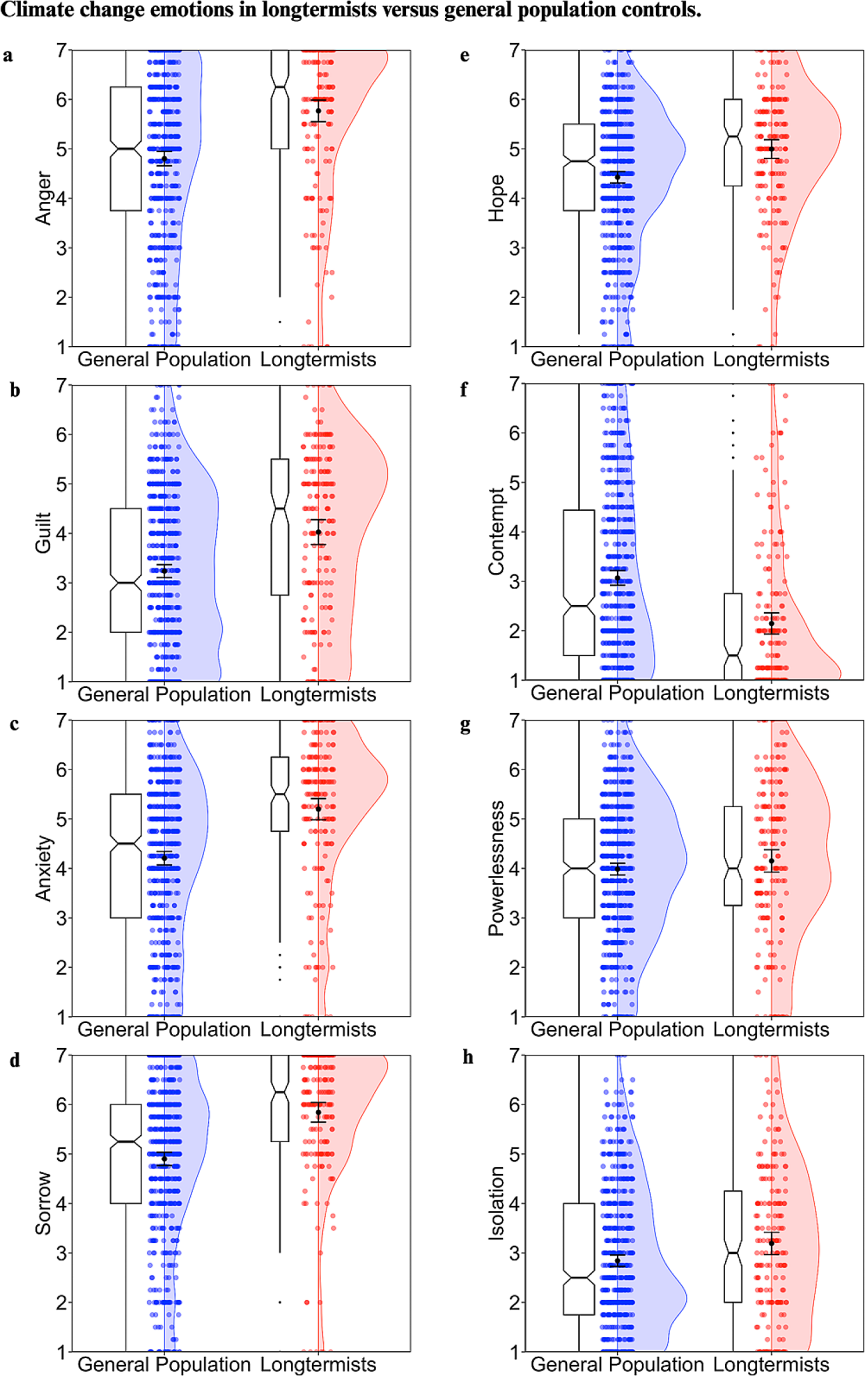
Plots depicting anger (**a**), guilt (**b**), anxiety (**c**), sorrow (**d**), hope (**e**), contempt (**f**), powerlessness (**g**), and isolation (**h**) for longtermists and general population controls. Ratings were made on a scale from 1 to 7 and averaged to form composite measures. Colored dots correspond to individual data points and are jittered for readability, with split violin plots overlaid to show the relative distribution of scores across populations. Error bars depict ± 1.96*SEM. Notched boxplots are included, with notches depicting a confidence interval around the median with a value of +/- 1.58*IQR/sqrt(n)

Supporting our hypothesis, longtermists scored significantly higher in anger, guilt, anxiety and sorrow, but importantly, they also reported significantly higher hope about climate change than non-longtermists. Further, they expressed significantly less contempt. Contrary to our hypothesis, no significant difference was noted for powerlessness. For isolation, we had contrasting hypotheses. The results suggested that longtermists felt more isolation compared to controls, possibly due their elevated concern for the issue relative to the rest of the population (See Table 2; Fig. 2).

#### Coping

**Fig. 3:**
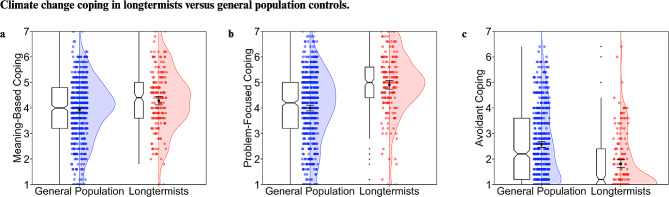
Plots depicting meaning-based (**a**), problem-focused (**b**), and avoidant coping for longtermists and general population controls. Ratings were made on a scale from 1 to 7 and averaged to form composite measures. Colored dots correspond to individual data points and are jittered for readability, with split violin plots overlaid to show the relative distribution of scores across populations. Error bars depict ± 1.96*SEM. Notched boxplots are included, with notches depicting a confidence interval around the median with a value of +/- 1.58*IQR/sqrt(n)

Supporting our hypothesis, longtermists scored significantly higher in meaning-based and problem-focused coping, and significantly lower in avoidant coping compared to non-longtermists (see Table 2; Fig. 3).

**Table 2:** Independent sample t-tests comparing longtermists to non-longtermists on all outcomes

Outcome	Non-longtermists		Longtermists					
	*M*	*SD*	*M*	*SD*	*t*	*df*	*p*	*d*
Legacy Concerns	4.86	1.34	5.57	1.25	6.46	769	< 0.001	0.55
ECAS	4.05	1.33	4.64	1.32	5.34	769	< 0.001	0.45
Problem-Focused Coping^†^	4.00	1.38	4.91	1.17	8.80	358.57	< 0.001	0.71
Avoidant Coping^†^	2.57	1.40	1.82	1.09	7.60	391.93	< 0.001	0.60
Meaning-Based Coping	3.94	1.18	4.28	1.15	3.53	769	< 0.001	0.30
Contempt^†^	3.07	1.81	2.15	1.49	6.94	370.94	< 0.001	0.55
Sorrow^†^	4.90	1.65	5.84	1.37	7.72	365.68	< 0.001	0.62
Anxiety^†^	4.21	1.69	5.20	1.48	7.66	346.66	< 0.001	0.62
Guilt	3.24	1.61	4.03	1.74	5.71	769	< 0.001	0.47
Powerlessness	3.99	1.45	4.15	1.58	1.33	769	0.185	0.11
Hope	4.43	1.43	5.00	1.30	4.81	769	< 0.001	0.42
Anger^†^	4.80	1.80	5.77	1.53	7.17	358.7	< 0.001	0.59
Isolation^†^	2.84	1.44	3.19	1.57	2.7	287.8	0.007	0.23

Note. † Satterthwaite approximation was employed to account for unequal variances between groups

### Legacy concerns and environmental cognitive alternatives as predictors of constructive coping and emotions

As shown in Table 3, and as hypothesized, both legacy concerns and ECAS related to more constructive coping, hope, anger, guilt, anxiety and sorrow about climate change, and less avoidant coping (only for ECAS), and contempt. We had competing hypotheses for isolation, but, given the results for longtermism, the pattern for the proposed mediators were in line the previous results, such that both legacy concerns and ECAS related to increased isolation. Importantly, these results remained significant, and in the same direction when controlling for age, gender, socioeconomic status and political ideology (see Table S2 in SOM).

**Table 3:** Linear regressions with legacy concerns and ECAS as predictors of all focal outcomes

Outcome	Adj. *R*^2^	Legacy Concern						ECAS				
		*b*	*β*	*p*	Lower 95% C.I.	Upper 95% C.I.		*b*	*β*	*p*	Lower 95% C.I.	Upper 95% C.I.
Contempt	0.07	-0.12	-0.09	0.013	-0.22	-0.03		-0.30	-0.22	< 0.001	-0.39	-0.20
Sorrow	0.11	0.22	0.18	< 0.001	0.13	0.31		0.28	0.23	< 0.001	0.19	0.37
Anxiety	0.14	0.26	0.21	< 0.001	0.18	0.35		0.30	0.24	< 0.001	0.22	0.39
Isolation	0.09	0.18	0.16	< 0.001	0.10	0.26		0.22	0.20	< 0.001	0.15	0.30
Guilt	0.10	0.27	0.22	< 0.001	0.18	0.36		0.21	0.17	< 0.001	0.12	0.30
Powerlessness	0.02	0.10	0.09	0.015	0.02	0.18		-0.17	-0.16	< 0.001	-0.25	-0.09
Hope	0.31	0.24	0.23	< 0.001	0.18	0.31		0.46	0.43	< 0.001	0.39	0.52
Anger	0.10	0.18	0.14	< 0.001	0.08	0.27		0.34	0.26	< 0.001	0.24	0.43
Problem-Focused Coping	0.38	0.35	0.34	< 0.001	0.29	0.41		0.43	0.42	< 0.001	0.37	0.49
Avoidant Coping	0.03	-0.06	-0.06	0.113	-0.14	0.01		-0.14	-0.14	< 0.001	-0.22	-0.07
Meaning-Based Coping	0.26	0.19	0.22	< 0.001	0.13	0.25		0.35	0.40	< 0.001	0.29	0.40

Note. Adj. = Adjusted

### Indirect effects

Based on the aforementioned theoretical rationale, and our pre-registered expectation for a significant indirect effect of longtermism on the different coping methods and emotional reactions to climate change, we estimated several indirect effects with longtermist identification as the predictor (binary variable; 1 = longtermist, 0 = non-longtermist), legacy concerns and ECAS as parallel mediators, and each outcome of interest as the focal outcome in each model. We used the PROCESS Macro [84], Model 4 (mediation with parallel mediators), with 10,000 bootstrapped samples.

In all models (see Table 4), longtermist identification predicted significantly higher legacy concerns (*b* = 0.72, 95% C.I. [0.50, 0.94], *R*^2^ = 0.05) and ECAS (*b* = 0.60, 95% C.I. [0.38, 0.82], *R*^2^ = 0.04). Even after controlling for legacy concerns and ECAS, being a longtermist predicted significantly higher scores for problem-focused coping, anger, guilt, sorrow, and anxiety for climate change, and significantly lower scores for contempt and avoidant coping, suggesting that legacy concerns and ECAS only partially mediate the effect of longtermism for these outcomes. However, for meaning-based coping and hope, the effect of longtermism was not significant after including the mediators. In turn, higher legacy concerns and ECAS related to increased problem-focused and meaning-based coping, hope, guilt, anger, isolation, anxiety, and sorrow, while ECAS specifically also related to decreased avoidant coping, and contempt. For powerlessness, legacy concerns had a positive association while ECAs a negative. Finally, significant indirect effects for both legacy concerns and ECAS emerged for problem-focused and meaning-based coping, anger, hope, powerlessness, guilt, isolation, anxiety, and sorrow. ECAS also mediated the effect of longtermism for avoidant coping and contempt. Results were for the most part consistent after accounting for the aforementioned demographic covariates, as all indirect effects except for the effect via ECAS for avoidant coping, remained significant and in the same direction (see Table S3 in SOM).

**Table 4:** Indirect effects test with longtermist identification as the exogenous variable, legacy concerns and ECAS as parallel mediators

Outcome	*R* ^2^	Effect of Longtermism	Effect of Legacy Concerns	Effect of ECAS	Indirect effect via Legacy Concerns	Indirect effect via ECAS
		*b* [95% C.I.]	*b* [95% C.I.]	*b* [95% C.I.]	*b* [95% C.I.]	*b* [95% C.I.]
Problem-focused coping	0.40	0.43 [0.25, 0.62]	0.32 [0.26, 0.38]	0.41 [0.35, 0.47]	0.23 [0.15, 0.32]	0.25 [0.15, 0.35]
Avoidant coping	0.07	− 0.67 [-0.89, − 0.44]	− 0.02 [-0.10, 0.05]	− 0.11 [-0.19, − 0.04]	− 0.02 [-0.07, 0.03]	− 0.07 [-0.13, − 0.02]
**Meaning-based coping**	0.26	0.01 [-0.17, 0.18]	0.19 [0.13, 0.25]	0.35 [0.29, 0.40]	0.14 [0.08, 0.20]	0.21 [0.12, 0.29]
Anger	0.13	0.68 [0.39, 0.96]	0.14 [0.04, 0.23]	0.31 [0.22, 0.40]	0.10 [0.03, 0.18]	0.18 [0.10, 0.29]
**Hope**	0.31	0.13 [-0.07, 0.33]	0.24 [0.17, 0.30]	0.45 [0.38, 0.52]	0.17 [0.10, 0.25]	0.27 [0.16, 0.38]
Powerlessness	0.03	0.21 [-0.04, 0.46]	0.09 [0.01, 0.17]	− 0.18 [-0.26, − 0.10]	0.06 [0.00, 0.13]	− 0.11 [-0.18, − 0.04]
Guilt	0.12	0.50 [0.23, 0.77]	0.24 [0.15, 0.33]	0.19 [0.10, 0.28]	0.17 [0.10, 0.26]	0.11 [0.05, 0.19]
Isolation	0.09	0.09 [-0.15, 0.33]	0.17 [0.09, 0.25]	0.22 [0.14, 0.30]	0.12 [0.06, 0.20]	0.13 [0.07, 0.21]
Anxiety	0.16	0.66 [0.40, 0.93]	0.22 [0.14, 0.31]	0.28 [0.19, 0.36]	0.16 [0.08, 0.25]	0.16 [0.09, 0.26]
Sorrow	0.14	0.66 [0.40, 0.92]	0.18 [0.10, 0.27]	0.25 [0.17, 0.34]	0.13 [0.06, 0.22]	0.15 [0.08, 0.24]
Contempt	0.10	− 0.70 [-0.99, − 0.41]	− 0.08 [-0.18, 0.02]	− 0.27 [-0.36, − 0.17]	− 0.06 [-0.14, 0.01]	− 0.16 [-0.25, − 0.08]

## Discussion

As climate change-related threats become more pronounced [1–4], researchers are now extending their focus beyond the physical environment to examine their impact on the mental health of individuals within it [5–11]. This research highlights not only that climate change impacts mental health, but that resulting coping strategies and emotional reactions are a critical driver of pro-environmental engagement. Despite extensive research demonstrating that intergenerational concern, as assessed by high scores on the Longtermism Beliefs Scale (LBS) [22], correlates with pro-environmental engagement [23, 69] and forward-thinking attitudes and behaviors aimed at safeguarding the well-being of future generations [20–24, 34], the precise impact of intergenerational concern on coping strategies and emotional reactions to climate change remains a critically unexplored area of study [masked for review]. The present study represents a first pass at testing the novel hypothesis that intergenerational concern serves the adaptive function of not only promoting attitudes and actions towards mitigating the deleterious effects of climate change on the natural environment, but also promoting proactive coping strategies towards mitigating its adverse effects on psychological well-being.

In this study, we delved into the climate change-related coping strategies [5–7, 9] and emotional reactions [8, 68] of empirically-identified longtermists, individuals who exhibit exceptionally strong intergenerational concern. Our findings revealed that longtermists tend to employ problem-focused (i.e., directing one’s effort and attention directly toward actively addressing the specific problem or stressor they are facing) and meaning-based (i.e., finding or creating meaning and purpose in difficult or challenging situations) coping strategies more frequently while relying less on avoidant coping strategies (i.e., engaging in efforts to distance oneself from a stressor) when confronted with climate change-related challenges. Intriguingly, despite experiencing emotions such as guilt, anger, sorrow, and a sense of isolation in response to climate change, longtermists also reported feeling a distinct sense of hope about the future, aligning with work which underscores the utility of emotional complexity in driving collective action [85].

The emotional responses we observed were in line with what we would expect for a group of people characterized by robust concern for the distant future. Having feelings about climate change (regardless of the valence) is indicative of involvement with the issue rather than avoidance. Longtermists care about the issue of climate change, and that care and concern manifest in their emotional engagement. While previous research has suggested that there is a level of emotional response needed to motivate action, experiencing worry too strongly could result in avoidance [75]. Yet, even among those who are experiencing strong negative emotions, maintaining a sense of hope and being able to make meaning represent one way of safeguarding well-being [73]. Longtermists appear to be especially good at maintaining hope. However, understanding how and why longtermists foster hope, and the intricate relationships between different emotions over time requires further study.

These observed patterns in coping and emotional responses were further elucidated by two key factors. First, longtermists’ heightened concerns about the legacy they will leave behind played a crucial role in driving their problem-focused and meaning-based coping strategies. These findings align with an extensive body of literature linking legacy motivations to proenvironmental attitudes and action [25, 35, 36, 61, 61–63, 86, 87] as well as intergenerational concern [20–22, 34]. Yet the present findings build upon this existing literature by demonstrating that legacy motivations simultaneously mitigate the impact of climate change on well-being. Second, longtermists’ enhanced ability to generate alternative solutions and perspectives regarding environmental issues, referred to as environmental cognitive alternatives (ECAs), contributed significantly to their positive outlook and adaptive coping mechanisms in the face of climate change. Similar to legacy motivations, ECAs have been shown to predict pro-environmental and otherwise farsighted attitudes and actions [26, 56]. Nonetheless, the present study is the first to connect this factor to climate-related coping and emotionality.

In summary, the present findings show that individuals with a strong sense of intergenerational concern experience a mix of emotions when it comes to climate change. They often grapple with feelings of anger, guilt, and anxiety in response to the climate crisis. However, in contrast to these negative emotions, they also maintain a sense of hope about the future, taking direct actions to address the challenges posed by climate change (problem-focused coping), actively engaging with the issue and seeking solutions. Simultaneously, they find meaning and purpose in their efforts, not losing faith in others or humanity as a whole (meaning-focused coping). Importantly, they do not resort to avoidant coping, which involves avoiding or ignoring the issue altogether. Instead, they confront climate change head-on, driven by their ability to envision of a more sustainable future and strong desire to leave behind a positive and lasting legacy. By instilling values that highlight intergenerational concern as a key priority, we may be able not only to increase pro-climate action, but also to help individuals actively and constructively cope with changes produced by climate change.

### Limitations and future directions

While the current study represents an initial exploration of the positive relationship that intergenerational concern has with climate-related coping and emotional responses, it is important to acknowledge several limitations and areas that warrant further investigation and exploration. For instance, the analyses in the current study are correlational, which means that the directionality and causal nature of the relationships under examination remain subject to further investigation. On the one hand, it is possible that intergenerational concern precedes and influences coping strategies and emotional responses to climate change. It is equally plausible that individuals’ coping mechanisms and emotional reactions to climate challenges may, in turn, shape the level of concern they feel toward safeguarding future generations from these threats. These potential causal relationships should be explored in future research that directly manipulates intergenerational concern. Previous research has demonstrated that low-cost and short-duration interventions can effectively promote intergenerational attitudes and actions [23, 34, 36, 61].

Furthermore, future research could consider applying the current framework within a longitudinal context. Given that coping mechanisms are designed to alleviate negative emotional states, it is reasonable to hypothesize that the proactive coping strategies used by individuals with heightened intergenerational concern might effectively mitigate the feelings of anger, guilt, and anxiety they experience in response to climate challenges over time. Longitudinal studies could provide valuable insights into the evolving dynamics of coping and emotional reactions in relation to climate change. Notably, while this study has shed light on the connections between intergenerational concern, coping strategies, and emotional responses, it has yet to establish a direct link between coping mechanisms and climate actions. Future research endeavors may bridge this gap by investigating how specific coping strategies translate into concrete pro-environmental behaviors and actions, providing a more comprehensive understanding of the interplay between emotional reactions, coping, and climate-related engagement.

Another area ripe for further investigation is the variance that persists between intergenerational concern and the outcomes. While our study has uncovered evidence suggesting that legacy concerns and environmental cognitive alternatives (ECAs) account for a portion of the variance in these relationships, there remains unexplained variance that calls for deeper exploration. Future research can explore additional factors and mechanisms that contribute to the complex relationship between intergenerational concern and outcomes related to coping and emotional reactions, offering a more comprehensive understanding of these dynamics.

As an example, longtermists exhibit heightened levels of future self-continuity, indicating a strong sense of identity with their future selves, and tend to carefully contemplate the consequences of their actions [88]. These variables are well-established predictors of forward-thinking behaviors aimed at safeguarding not only one’s own future but also the future well-being of others [89–99, 99]. Moreover, longtermists engage in greater utopian thinking, and report a greater vividness when envisioning the distant future compared to general population controls, characteristics that contribute to intergenerational and pro-environmental concerns [20, 54, 55, 58, 59, 69]. Given the relevance of these additional factors, they merit consideration as candidate mechanisms that could help bridge the gap between intergenerational concern and coping strategies, as well as emotional reactions in the context of climate change.

Lastly, it’s essential to acknowledge that the current study is based on a sample from the United States. Future research endeavors should aim to replicate and extend the findings presented here in an international context. Examining these relationships across diverse cultural and geographic settings may help uncover valuable insights into the universality or cultural specificity of the observed patterns, contributing to a more comprehensive understanding of intergenerational concern and its implications for climate-related coping and emotional responses on a global scale. For instance, prior research has found that countries with cultures embodying a long-term orientation and with greater intergenerational solidarity score higher on numerous metrics of environmental performance [100]. Moreover, citizens of these countries are more concerned about the negative effects of climate change. What stands to be addressed in future research is whether national differences in temporal perspective predict variation in coping with and emotional reactions to climate change, in turn facilitating greater proenvironmental engagement.

## Conclusion

Climate change impacts are already occurring but will be catastrophic for future generations without intervention. Longtermists, and those with high levels of intergenerational concern more broadly, represent a group of people who are particularly concerned about future generations. This intergenerational concern does not appear to paralyze them or prevent engagement with the issue; in fact, we find that longtermists are able to feel hopeful in spite of the challenges and engage in proactive coping strategies to address climate change.

### Electronic supplementary material

Below is the link to the electronic supplementary material.

Supplementary Material 1

## Data Availability

Data have been made publicly available on the OSF (https://osf.io/ndqz2/?view_only=adef6f1b8aa44aeeac90645feb2309f4).
