# Supplementary material for: Intergenerational concern relates to constructive coping and emotional reactions to climate change via increased legacy concerns and environmental cognitive alternatives
Source: BMC Psychol. 2024 Apr 2;12:182. doi: 10.1186/s40359-024-01690-0 (PMC10986099; doi:10.1186/s40359-024-01690-0)
Supplement: Supplementary file 1 — Supplementary Material 1 [file 40359_2024_1690_MOESM1_ESM.docx]

Intergenerational Concern Relates to Constructive Coping and Emotional Reactions to Climate Change via Increased Legacy Concerns and Environmental Cognitive Alternatives

**Supplementary Analyses**

**Table S1.**

*Results for comparisons of longtermists and non-longtermists controlling for age, gender, SES and political ideology.*

| Outcome | Adjusted R^2^ | b | β | p | Lower 95% CI | Upper 95% CI |
| --- | --- | --- | --- | --- | --- | --- |
| Contempt | 0.35 | -0.63 | -0.15 | <.001 | -0.88 | -0.39 |
| Sorrow | 0.25 | 0.72 | 0.19 | <.001 | 0.48 | 0.96 |
| Anxiety | 0.24 | 0.80 | 0.20 | <.001 | 0.55 | 1.05 |
| Isolation | 0.02 | 0.31 | 0.09 | 0.012 | 0.07 | 0.56 |
| Guilt | 0.10 | 0.71 | 0.18 | <.001 | 0.44 | 0.98 |
| Powerlessness | 0.05 | 0.08 | 0.02 | 0.527 | -0.16 | 0.32 |
| Hope | 0.14 | 0.51 | 0.15 | <.001 | 0.28 | 0.73 |
| Anger | 0.28 | 0.72 | 0.17 | <.001 | 0.46 | 0.97 |
| Problem-Focused Coping | 0.17 | 0.85 | 0.26 | <.001 | 0.64 | 1.07 |
| Avoidant Coping | 0.32 | -0.53 | -0.16 | <.001 | -0.72 | -0.34 |
| Meaning-Based Coping | 0.09 | 0.38 | 0.14 | <.001 | 0.19 | 0.57 |
| Legacy Concerns | 0.10 | 0.76 | 0.24 | <.001 | 0.54 | 0.97 |
| ECAS | 0.08 | 0.59 | 0.19 | <.001 | 0.38 | 0.81 |

**Table S2.**

*Linear regressions with legacy concerns and ECAS as predictors of all focal outcomes, controlling for age, gender, SES and political ideology.*

|  |  | Legacy Concern | | | | |  | ECAS | | | | |
| --- | --- | --- | --- | --- | --- | --- | --- | --- | --- | --- | --- | --- |
| Outcome | Adj. R^2^ | b | β | p | Lower 95% CI | Upper 95% CI |  | b | β | p | Lower 95% CI | Upper 95% CI |
| Contempt | 0.37 | -0.15 | -0.12 | <.001 | -0.24 | -0.07 |  | -0.18 | -0.14 | <.001 | -0.26 | -0.10 |
| Sorrow | 0.31 | 0.25 | 0.21 | <.001 | 0.17 | 0.33 |  | 0.22 | 0.18 | <.001 | 0.14 | 0.30 |
| Anxiety | 0.32 | 0.29 | 0.23 | <.001 | 0.21 | 0.37 |  | 0.23 | 0.18 | <.001 | 0.15 | 0.31 |
| Isolation | 0.10 | 0.20 | 0.18 | <.001 | 0.12 | 0.27 |  | 0.20 | 0.19 | <.001 | 0.12 | 0.28 |
| Guilt | 0.15 | 0.28 | 0.22 | <.001 | 0.19 | 0.36 |  | 0.18 | 0.14 | <.001 | 0.09 | 0.26 |
| Powerlessness | 0.08 | 0.12 | 0.11 | 0.003 | 0.04 | 0.20 |  | -0.20 | -0.18 | <.001 | -0.28 | -0.12 |
| Hope | 0.36 | 0.24 | 0.23 | <.001 | 0.17 | 0.30 |  | 0.41 | 0.39 | <.001 | 0.34 | 0.47 |
| Anger | 0.33 | 0.21 | 0.16 | <.001 | 0.13 | 0.29 |  | 0.24 | 0.18 | <.001 | 0.16 | 0.33 |
| Problem-Focused Coping | 0.44 | 0.34 | 0.33 | <.001 | 0.28 | 0.40 |  | 0.40 | 0.39 | <.001 | 0.34 | 0.46 |
| Avoidant Coping | 0.31 | -0.09 | -0.09 | 0.005 | -0.16 | -0.03 |  | -0.07 | -0.07 | 0.036 | -0.14 | 0.00 |
| Meaning-Based Coping | 0.29 | 0.16 | 0.19 | <.001 | 0.11 | 0.22 |  | 0.33 | 0.38 | <.001 | 0.28 | 0.39 |

**Note.** Adj. = Adjusted.

**Table S3.**

*Indirect effects test with longtermist identification as the exogenous variable, legacy concerns and ECAS as parallel mediators, controlling for age, gender, SES and political ideology.*

|  |  | Effect of Longtermism | Effect of Legacy Concerns | Effect of ECAS | Indirect effect via Legacy Concerns | Indirect effect via ECAS |
| --- | --- | --- | --- | --- | --- | --- |
| Outcome | R^2^ | b [95% C.I.] | b [95% C.I.] | b [95% C.I.] | b [95% C.I.] | b [95% C.I.] |
| Problem-focused coping | 0.45 | .39  [.21, .57] | .31  [.25, .37] | .39  [.33, .45] | .23  [.16, .32] | .23  [.14, .33] |
| Avoidant coping | 0.33 | -.45 [-.64, -.25] | -.06  [-.13, .00] | -.05  [-.12, .01] | -.05  [-.10, -.01] | -.03  [-.07, .01] |
| Meaning-based coping | 0.30 | .06  [-.11, .24] | .16  [.10, .22] | .33  [.27, .39] | .12  [.07, .18] | .20  [.12, .28] |
| Anger | 0.34 | .44  [.19, .70] | .18  [.10, .27] | .22  [.14, .31] | .14  [.07, .22] | .13  [.07, .21] |
| Hope | 0.37 | .09  [-.11, .29] | .23  [.17, .30] | .41  [.34, .47] | .18  [.11, .25] | .24  [.14, .35] |
| Powerlessness | 0.09 | .11  [-.13, .36] | .11  [.03, .19] | -.21  [-.29, -.12] | .09  [.02, .16] | -.12  [-.20, -.06] |
| Guilt | 0.17 | .42  [.16, .69] | .25  [.16, .34] | .16  [.07, .25] | .19  [.11, .28] | .09  [.03, .17] |
| Isolation | 0.11 | .05  [-.20, .29] | .19  [.11, .27] | .20  [.12, .28] | .14  [.09, .22] | .12  [.06, .20] |
| Anxiety | 0.33 | .47  [.23, .71] | .26  [.18, .34] | .21  [.13, .29] | .20  [.12, .29] | .13  [.06, .20] |
| Sorrow | 0.33 | .43  [.20, .66] | .22  [.14, .30] | .20  [.12, .28] | .17  [.09, .26] | .12  [.06, .20] |
| Contempt | 0.39 | -.44  [-.68, -.19] | -.13 [-.21, -.04] | -.17  [-.25, -.08] | -.10  [-.17, .03] | -.10  [-.17, -.04] |

**Note.** The effects of longtermism identification on legacy concerns (*b* = 0.76, 95% C.I. [.54, .97]) and ECAS (*b* = 0.49, 95% C.I. [.38, .81]) were also significant after controlling for all covariates.
